# When 25% Feels Like 100%: Confronting Recurrent Cystic Fibrosis Risk Across Consecutive Pregnancies

**DOI:** 10.7759/cureus.97373

**Published:** 2025-11-20

**Authors:** Sujoy Dasgupta, Dipanjana Datta, Soumyajit Pal, Kusagradhi Ghosh, Shyamal Sett

**Affiliations:** 1 Reproductive Medicine, Genome - The Fertility Centre, Kolkata, IND; 2 Genetics, Institute of Fetal Medicine, Kolkata, IND; 3 Obstetrics and Gynecology, KPC Medical College and Hospital, Kolkata, IND; 4 Fetal Medicine, Institute of Fetal Medicine, Kolkata, IND; 5 Obstetrics and Gynecology, Zenith Super Specialist Hospital, Kolkata, IND

**Keywords:** chorionic villus sampling, cystic fibrosis in india, cystic fibrosis transmembrane conductance regulator (cftr) protein, f508del mutation, multiplex ligation-dependent probe amplification, next generation sequencing (ngs), preimplantation genetic testing, recurrence in consecutive pregnancies, targeted exome sequencing

## Abstract

Cystic fibrosis (CF) is the most common autosomal recessive lethal disorder worldwide caused by mutations in the CF transmembrane conductance regulator (CFTR) gene, which represents conditions with variable expressivity and penetrance. CF is not uncommon in India, but with a more genetically heterogeneous mutation spectrum. We present a case where a couple came for preconception counselling after they lost their baby at seven months of age because of CF. Initial targeted exome sequencing of the baby suggested a homozygous pathogenic variant of F508del. The father was a carrier of the F508 variant, while the mother tested negative for this variant. Because of this discrepancy, subsequent testing, multiple ligation-dependent probe amplification (MLPA) was performed, which revealed the mother carried a heterozygous multi-exon deletion (exons 4-11). Re-analysis of preserved tissue of the baby using MLPA diagnosed compound heterozygosity for F508del and the exon 4-11 deletion. This case underscores the importance of combining sequence variant detection with copy number variation (CNV) analysis in CFTR testing to avoid misinterpretation of zygosity. None of the parents had any stigmata of CF-related disease, including congenital bilateral absence of the vas deferens (CBAVD). After discussion, the couple decided on preimplantation genetic testing for monogenic conditions (PGT-M), which yielded a single embryo harboring a compound heterozygous mutation (F508del and exons 4-11). Subsequently, the mother conceived naturally, but prenatal diagnosis by chorionic villus sampling (CVS) found the presence of the same compound heterozygous mutations in the fetus, for which the pregnancy was terminated. Fortunately, in the subsequent spontaneous conception, CVS revealed that the fetus was only heterozygous for exons 4-11, establishing its carrier status. Pregnancy eventually ended in the live birth of the baby, who is now doing well at 20 months of age. Parents, in this case, had a long journey, where the risk of 25% in every pregnancy seemed to be difficult for them to carry on because it recurred three consecutive times in the fetus/embryo(s). This illustrates how statistical probabilities, though clinically accurate, can feel meaningless to those enduring repeated loss, and underscores the profound psychological toll of autosomal recessive disorders. Psychological support, genetic counselling, and ensuring informed decision-making are crucial for parents coping with the psychological impact of having a child with a rare disease. In this case, it is the perseverance of the couple that finally led to a successful outcome. Complexity of genetic analysis and counselling, recurrence three consecutive times, absence of CBAVD in the man despite carrying the CFTR mutation, and finally a successful outcome prompted us to report this case.

## Introduction

Cystic fibrosis (CF) is a life-threatening genetic disorder caused by mutations in the CF transmembrane conductance regulator (CFTR) gene located in chromosome 7 [1-3]. This gene encodes an epithelial ion channel responsible for bicarbonate and chloride transfer [1-3]. Therefore, the mutations result in impaired chloride transport and defective hydration and clearance [1-3], leading to the production of thick, sticky mucus that clogs the lungs and the pancreas [1-3]. It is the most common autosomal recessive lethal disorder worldwide, with the highest prevalence observed in the Caucasian population [2,3]. CF affects nearly 100,000 people worldwide [1].

Symptoms of CF vary widely but commonly include chronic cough, recurrent lung infections, poor growth, and digestive issues, which can eventually lead to exocrine pancreatic insufficiency, diabetes, chronic lung disease, and hepatic dysfunction [1-3]. Men affected with CF may have defective sperm transport because of congenital bilateral absence of the vas deferens (CBAVD), leading to obstructive azoospermia [2,3]. Women affected with CF often have fertility issues because of poor nutrition and abnormally thick cervical mucus [2]. Because of poor nutrition, bone mineral density is affected, leading to kyphoscoliosis, pathological fractures, and clubbed fingers [2]. While there is no cure for CF, advancements in treatment and care have significantly improved life expectancy and quality of life for individuals living with the condition [1,2]. Early diagnosis, comprehensive management strategies, and ongoing medical care are essential in managing CF and improving outcomes for patients [1-3].

CF is inherited in an autosomal recessive manner, meaning that it is caused by inheriting two copies of a mutated gene, one from each parent [1-3]. When both parents are carriers of the mutated gene, there is a 25% chance with each pregnancy that the child will inherit both mutated alleles and manifest the disorder [2]. In such a case, the child will develop classical CF. The most common mutation in the Caucasian population is F508del (deletion of phenylalanine at the 508 position resulting from the deletion of three nucleotides, c.1521_1523delCTT) [2,3].

In some individuals with a single CFTR pathogenic variant, CFTR-related disorders (CFTR-RDs) happen, which include CBAVD, idiopathic chronic pancreatitis, bronchiectasis, and susceptibility to certain infections [3]. This means CFTR mutations do not strictly follow the “all or none” patterns of manifestation, unlike other autosomal recessive genetic disorders, but rather represent conditions with variable expressivity and penetrance [3]. CBAVD is more common in individuals with residual CFTR activity, leading to preserved pancreatic function [3]. The 5T allele is often associated with CABVD, commonly seen in heterozygous men, possibly because of increased sensitivity of vasa to CFTR dysfunction [3]. However, many men with a single CFTR pathogenic variant may have normal bilateral vasa [3].

In the case of CF, like any other autosomal recessive disorder, genetic counseling and prenatal testing are crucial for couples to understand the risks and make informed reproductive decisions [4,5].

Here we present a case report where a couple experienced CF three consecutive times: first time in the infant who succumbed because of CF, second time in the embryo produced in vitro, and third time in the fetus after prenatal invasive testing. However, finally, they had a successful live birth with a baby who was only heterozygous for the CFTR mutation.

## Case presentation

In June 2021, a 34-year-old woman in a non-consanguineous marriage came to our clinic with her 34-year-old partner as they wanted to conceive again. She had undergone laparoscopic left ovarian cystectomy and adhesiolysis for dysmenorrhea in 2018, and the histopathology revealed endometriosis. Apart from this, none of the partners had any significant medical or surgical history. She had a regular menstrual cycle and had no dysmenorrhea after previous laparoscopic surgery. None of them were addicted to smoking, alcohol, or recreational drugs. None of them had any family members affected with any known genetic disorders. Her first pregnancy was terminated surgically in 2018 because of contraception failure. In her second pregnancy, she underwent an emergency cesarean section in November 2020 at 32 weeks of pregnancy because of severe antepartum hemorrhage caused by placenta previa. The skin incision was midline vertical, followed by a low vertical incision in the uterus. Significant pelvic adhesion was encountered during the surgery. A live female baby was delivered, who was later diagnosed with CF.

The baby had clinical manifestations of failure to thrive, doll-like facies, recurrent chest infections, recurrent aspiration of milk, and edema. Initial targeted exome sequencing suggested a homozygous pathogenic variant in the CFTR gene at exon 11 (c.1521_1523del, also known as F508del). However, upon re-analysis and use of multiple ligation-dependent probe amplification (MLPA), the infant was found to be compound heterozygous for F508del and a multi-exon deletion spanning exons 4 to 11. Unfortunately, the infant passed away at seven months of age in June 2021. The couple was referred for genetic counseling to understand their reproductive risks and options for future pregnancies.

Investigations

The couple was referred for genetic counseling to understand their chances of having a healthy child. In July 2021, they underwent genetic counseling and carrier screening. The father was identified as a carrier of the ΔF508 (F508del) variant in the CFTR gene. The mother, however, tested negative for this variant, despite the initial report on their affected child indicating a homozygous F508del mutation. This discrepancy suggested the possibility of a false-positive finding for homozygosity in the earlier report on the child. Given that the child exhibited a classical CF phenotype, further analysis was warranted. CFTR-MLPA testing was subsequently performed, which revealed that the mother carried a heterozygous deletion spanning exons 4-11 of the CFTR gene. Re-evaluation of the stored deoxyribonucleic acid (DNA) sample of the index child confirmed these findings; the child indeed had a genotype consistent with compound heterozygosity for F508del and the exon 4-11 deletion. This case underscores the importance of combining sequence variant detection with copy number variation (CNV) analysis in CFTR testing. Given the gene’s complex architecture with pseudogenes and recurrent structural variant hotspots, integrating both approaches ensures diagnostic accuracy and prevents misinterpretation of zygosity in CF testing.

The couple was in trauma and wanted to conceive a healthy baby. They were recommended for prenatal diagnosis. Alternative reproductive options of preimplantation genetic testing for monogenic conditions (PGT-M) or gamete donation were also discussed.

As they were concerned about the female's age (as she was 35 years old by this time), they decided on an in vitro fertilization (IVF) cycle with the intention of PGT-M. Investigations revealed that she had a serum anti-Müllerian hormone (AMH) level of 2.8 ng/ml and an antral follicle count of 7-8 in each ovary. He was found to have normozoospermia and had both vasa palpable during scrotal examination with normal testicular size bilaterally. In May 2022, the couple started the first IVF cycle, but it was cancelled five days after stimulation because she developed a severe COVID-19 infection with respiratory illness. In August 2022, the fertility treatment was resumed, using an antagonist protocol. The drugs used were subcutaneous injections of human menopausal gonadotrophin (Menotas HP, Intas Pharmaceuticals Limited®, India) at 225 IU/day for 10 days, subcutaneous injections of cetrorelix (Cetrolix, Intas Pharmaceuticals Limited®, India) at 0.25 mg/day for the last four days, and subcutaneous injections of human chorionic gonadotrophin (Coriosurge HP, Intas Pharmaceuticals Limited®, India) at 5000 IU as a final maturation trigger. A total of eight oocytes were retrieved, out of which five were in the metaphase-2 stage. However, following intracytoplasmic sperm injection (ICSI), only one grade 4AA blastocyst was formed on day 5, and a trophectoderm biopsy was carried out, followed by freezing that embryo. PGT-M indicated that the embryo harbored both the CFTR variants and hence was at risk of disease manifestation, thus not suitable for implantation. Consequently, the embryo was not transferred.

Outcome and follow-up

In December 2022, the couple returned after conceiving naturally, but prenatal diagnosis by chorionic villus sampling (CVS) revealed that the fetus harbored both the variants and was affected. The couple decided on surgical termination of the pregnancy due to poor prognosis.

In July 2023, another spontaneous pregnancy was evaluated, and prenatal diagnosis by CVS indicated that the fetus harbored a heterozygous CFTR variant from the mother and the normal wild-type variant from the father, thus establishing its carrier status.

**Figure 1:**
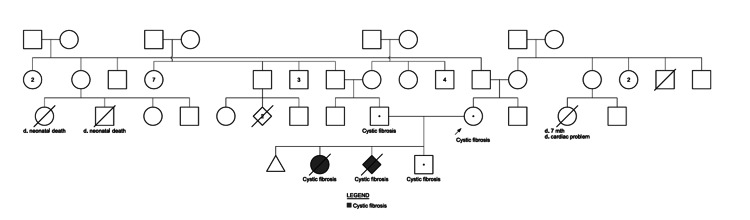
The pedigree shows multiple affected individuals consistent with an autosomal recessive pattern of inheritance Males are represented by squares and females by circles. Affected individuals with cystic fibrosis are indicated by shaded symbols (▧). Deceased individuals are marked with a diagonal slash (/), and the age or cause of death is noted below the symbol where known. Carriers identified through genetic testing are represented by a central dot (•). Neonatal deaths are indicated as “d. neonatal death,” and one infant who died at seven months due to a cardiac problem is annotated. The pedigree demonstrates recurrence of cystic fibrosis among the offspring of carrier couples across two generations, highlighting segregation of CF transmembrane conductance regulator (CFTR) variants within the extended family. The image is created by the authors of this study.

Pregnancy was uncomplicated. An elective cesarean section was planned at 39 completed weeks of gestation because of a previous classical section. As the placenta was in the posterior and upper portion of the uterus, lower segment caesarean section (LSCS) was possible through a transverse skin incision. Severe adhesions in the lower segment, with the bladder and omentum, were encountered, which were divided by sharp and blunt dissection. There were no other significant intra- or postoperative events encountered. The baby is now 20 months old, and he is doing well. Table 1 summarizes the CFTR variants identified in our case. The pedigree analysis is depicted in Figure 1.

**Table 1:** CFTR variants identified in our case CBAVD: congenital bilateral absence of the vas deferens; CF: cystic fibrosis; CFTR: CF transmembrane conductance regulator; CNV: copy number variation; CVS: chorionic villus sampling; MLPA: multiplex ligation-dependent probe amplification; PGT-M: preimplantation genetic testing for monogenic conditions; ICSI: intracytoplasmic sperm injection

Individual/Sample	Variant	Genomic Location (hg38, chr7)	Zygosity	dbSNP/ClinVar ID	Notes
Index child	c.1521_1523delCTT (F508del, p.Phe508del)	chr7:117,559,593_117,559,595del (3-bp deletion in exon 11)	Heterozygous (paternal)	dbSNP: rs113993960; ClinVar: VCV000007105.61	Initially misinterpreted as homozygous for ΔF508del, until MLPA clarified compound het status.
	Multi-exon deletion (exons 4–11)	chr7:117,105,838–117,559,651 (approx span, depending on breakpoint)	Heterozygous (maternal)	ClinVar: multiple entries (e.g., VCV000852457.1); no dbSNP (structural CNV)	Large multi-exon deletion confirmed by MLPA; recurrent hotspot CNV in CFTR.
Father	F508del (c.1521_1523delCTT)	chr7:117,559,593_117,559,595del	Heterozygous (carrier)	rs113993960 / VCV000007105	Asymptomatic carrier (Normozoospermia, no CBAVD).
Mother	Multi-exon deletion (exons 4–11)	chr7:117,105,838–117,559,651	Heterozygous (carrier)	CNV ClinVar entry (VCV000852457)	Asymptomatic carrier.
PGT-M embryo (ICSI, 2022)	F508del + multi-exon del	–	Compound heterozygous	Same as above	Affected embryo, not transferred.
Pregnancy, Dec 2022 (CVS)	F508del + multi-exon del	–	Compound heterozygous	Same as above	Affected fetus, the couple opted for termination.
Pregnancy, July 2023 (CVS)	Multi-exon deletion only (exons 4–11)	chr7:117,105,838–117,559,651	Heterozygous	VCV000852457	Carrier fetus, unaffected / subsequently healthy live birth.

## Discussion

India is a land where endogamy and consanguinity are common, leading to increased prevalence of such disorders due to the accumulation of deleterious traits across generations [5]. In the Indian scenario, the true prevalence of CF remains uncertain due to limited population-based data and underdiagnosis, but emerging evidence suggests it may be far more common than previously believed [6-16]. Early estimates, largely based on Diaspora data, placed the incidence between one in 10,000 to one in 40,000 births [7-9,13], while clinic-based studies in India suggested even lower figures of one in 43,000 to one in 100,000 births [7,9,13]. More recent carrier frequency studies, however, reported a CFTR carrier rate of one in 41, translating to an estimated disease incidence of one in 6,588 births, or nearly 3,600 new cases annually, given India’s high birth rate [16].

The F508del mutation is the most frequently reported pathogenic allele in Indian CF patients, accounting for 19-44% of cases (Table 2), but this proportion is much lower than in Western populations, reflecting a more genetically heterogeneous mutation spectrum in India [6-16].

**Table 2:** Indian CFTR/CF prevalence (selected publications) CF: cystic fibrosis; CFTR: CF transmembrane conductance regulator

First Author and Year	Sample (n)	Aim of the Study	Key Estimate (India)	Outcome/Comments
Kabra, 2003 [6]	74	Indian cohort ΔF508 frequency	19% of CF alleles	First Indian CF cohort; highlights very low F508del versus Western (~70%).
Kapoor, 2006 [7]	955 cord blood	Indian cohort carrier frequency of ΔF508	Carrier 1/238 → estimated prevalence 1/43,321–1/100,323	First population-based estimate; underestimation as only F508del tested.
Kabra, 2007 [8]	120	Review of Indian CFTR data	F508del = 19–34%	Notes delayed diagnosis, poor awareness, mutation heterogeneity.
Prasad, 2010 [9]	–	Review	ΔF508 is much less frequent than West	One of the first comprehensive Indian reviews; highlights underdiagnosis.
Ashavaid, 2012 [10]	–	Systematic review	Limited prevalence data (19-56% F508del mutation prevalence)	Calls for structured screening and better genotyping.
Sharma, 2015 [11]		Indian cohort mutation spectrum	F508del ~30%; (27% frequency) heterogeneous alleles	Notes CFTR panel from West misses Indian variants; CBAVD overlap.
Girisha, 2014 [12]		Editorial	F508del = 31.1%	Aggregated analysis; Indian F508del far below Western.
Mandal, 2015 [13]	–	Review	Incidence ~1/40,000	F508del mutation is more prevalent in Northern India than the Southern part.
Prasad, 2018 [14]		Review (South Asian data)	F508del = 19–44% in the South Asian population	Regional analysis across South Asia; still far lower than the West.
Varkki, 2024 [15]	120	Retrospective	27% of CF alleles	Very low F508del vs Western (~70%).
Farrell, 2025 [16]		Commentary	Incidence 1/7,000–1/12,000	Argues India’s true CF burden is much higher; calls for newborn screening rollout.

Other recurrent mutations reported in the Indian population include p․Ser549Asn, p.Gly542X, p.Trp1282X, c.1161delC, and p.Asn1303Lys (Table 3), with regional variation across the country [6,11-13]. Despite this knowledge, the diagnosis is often delayed or missed because of limited diagnostic resources, for which many affected children are diagnosed late, often after developing severe respiratory and gastrointestinal manifestations or malnutrition [6,8,13,15,16]. Furthermore, currently there is no nationwide newborn screening program for CF in India, and prevalence estimates remain variable [9,11,16].

**Table 3:** Indian CFTR variants beyond F508del CBAVD: congenital bilateral absence of the vas deferens; CF: cystic fibrosis; CFTR: CF transmembrane conductance regulator

Variant	Frequency/Detection in India	Associated Phenotype(s)	First Author and Year	Notes
p․Ser549Asn (S549N)	One of the most recurrent non-F508del variants in Indian CF patients	Classical CF, pancreatic insufficiency	Kabra, 2003 [6]	Found in the North Indian cohort; also seen in South Asia.
p.Gly542X (G542X)	Detected in multiple Indian series	Severe CF, early-onset	Kabra, 2003; Sharma, 2015 [6,11]	Known Mediterranean founder mutation; present in Indians at a lower frequency.
p.Trp1282X (W1282X)	Rare but reported in Indian CF patients	Severe CF, early childhood diagnosis	Kabra, 2003 [6]	Common in Ashkenazi Jewish; sporadic in India.
c.1161delC (frameshift)	Reported in Indian cases	Classical CF	Sharma, 2015 [11]	Frameshift, pathogenic.
p.Asn1303Lys (N1303K)	Reported in multiple Indian patients	Severe CF	Sharma, 2015 [11]	Common in global cohorts, present in India at low frequency.
p.Ile148Tyr (I148T)	Rare in Indian reports	CFTR-related disorder, variable phenotype	Prasad, 2010 [9]	Known in other populations, occasionally in the Indian spectrum.
p.Gly551Asp (G551D)	Rare in India	CF (gating mutation, Ivacaftor-responsive)	Sharma, 2015 [11]	Important pharmacogenetic implications if found.
p.Arg334Trp (R334W)	Rare	CFTR-related disorder	Sharma, 2015 [11]	Seen in mixed-ethnicity studies, including Indian patients.
Novel/unique variants	Multiple private/novel variants reported in Indian families	CF, CBAVD, atypical CF	Sharma, 2015; Girisha, 2014 [11,12]	Highlights mutation heterogeneity in Indian cohorts.

After her last termination in 2022, the female poignantly remarked: “Doctor, you said it’s 25%, but every time I have an affected fetus, whether it is a live birth, PGT-M, or CVS. For you it is 25%, but for me it is 100%.” This illustrates how statistical probabilities, though clinically accurate, can feel meaningless to those enduring repeated loss, and underscores the profound psychological toll of autosomal recessive disorders. While each pregnancy carries a 25% recurrence risk in CF, independent of prior outcomes, for the couple, this experience feels absolute, making prenatal diagnosis essential in every pregnancy [4].

Notably, neither partner exhibited any clinical “stigmata” of CFTR-related disorders; even the male partner had normally developed bilateral vasa. Thus, absence of phenotype does not rule out CFTR variants, reinforcing the importance of genetic testing [3].

The initial report suggested a homozygous mutation, but further evaluation revealed a larger CNV missed by exome analysis. Early exome platforms lacked sensitivity for CNVs, and in genes with pseudogenes like CFTR, the risk of false positives is high. Comprehensive evaluation, therefore, requires MLPA or next-generation sequencing (NGS) platforms with CNV detection capability [17,18]. Genetic counselling enabled CNV delineation via MLPA, ensuring accurate genotype determination, which is critical for reliable prenatal and preimplantation diagnosis [18]. More broadly, this emphasizes that exome sequencing alone cannot detect all deletions or duplications, making cascade testing in at-risk families essential [17]. Importantly, direct fetal testing without first confirming parental carrier status is not recommended, as it can lead to false positives (confusing carrier states with biallelic mutations) and expose the pregnancy to unnecessary iatrogenic risk [17,18]. Finally, counselling should be strengthened with resources such as the CFTR2 database, ethnicity-specific variant repositories, and the recommendations by the American College of Medical Genetics and Genomics (ACMG) on carrier screening [18]. Use of visual pedigree tools and provision of psychosocial support systems are equally vital to help families navigate both the medical and emotional complexities of recurrent genetic risk [17].

## Conclusions

A couple who have had a diagnosis of an inherited rare genetic disease like CF and lost a child to it requires ongoing support and tailored options within the constraints of cost. Parents, in this case, had a long journey, where the risk of 25% in every pregnancy seemed to be difficult for them to carry on because it recurred three consecutive times in the fetus/embryo(s). Each positive result in the fetus/embryo was a trauma they endured. Genetic counselling, accurate molecular diagnostics (including CNV evaluation), and access to psychological support are essential to support informed decisions and better outcomes. In our case, it is the perseverance of the couple that finally led to a successful outcome. Complexity of genetic analysis and counselling, recurrence three consecutive times, absence of CBAVD in the man despite carrying the CFTR mutation, and finally a successful outcome prompted us to report this case.
